# Advances in Copper-Based Biomaterials With Antibacterial and Osteogenic Properties for Bone Tissue Engineering

**DOI:** 10.3389/fbioe.2021.795425

**Published:** 2022-01-20

**Authors:** Qiudi Shen, Yansong Qi, Yangzhi Kong, Huricha Bao, Yifan Wang, Alideertu Dong, Haixia Wu, Yongsheng Xu

**Affiliations:** ^1^ College of Chemistry and Chemical Engineering, Inner Mongolia University, Hohhot, China; ^2^ Department of Orthopedics, Inner Mongolia People’s Hospital, Hohhot, China

**Keywords:** copper, biomaterials, antibacterial, osteogenic, bone tissue engineering

## Abstract

Treating bone defects coupled with pathogen infections poses a formidable challenge to clinical medicine. Thus, there is an urgent need to develop orthopedic implants that provide excellent antibacterial and osteogenic properties. Of the various types, copper-based biomaterials capable of both regenerating bone and fighting infections are an effective therapeutic strategy for bone tissue engineering and therefore have attracted significant research interest. This review examines the advantages of copper-based biomaterials for biological functions and introduces these materials’ antibacterial mechanisms. We summarize current knowledge about the application of copper-based biomaterials with antimicrobial and osteogenic properties in the prevention and treatment of bone infection and discuss their potential uses in the field of orthopedics. By examining both broad and in-depth research, this review functions as a practical guide to developing copper-based biomaterials and offers directions for possible future work.

## Introduction

Bone tissue is a mineralized, viscous, elastic connective tissue that plays an important role in the human body (Kasugai, 2003). As a rigid organ, bone not only supports and protects various other organs but also enables a range of activities (John et al., 2008). Bone tissue includes inorganic and organic components. The main inorganic component is hydroxyapatite, a mineral composed of calcium phosphate, and the main organic components are collagen and non-collagen fibers. The inorganic ingredients make bones strong, while collagen provides elasticity. As a living tissue, human bones continually engage in the processes of bone resorption and deposition, which are controlled by various cells: bone cells, osteoclasts, Gegenbaur cells, and lining cells. Bone cells are embedded in the bone matrix, while osteoclasts, Gegenbaur cells, and lining cells mainly exist on the bone surface and play an important role in bone turnover.

Bone defect refers to the absence of bone tissue at a specific anatomical location, which is generally the sequelae of high-intensity impact or trauma, tumor resection, infection, or revision surgery (Clarke, 2008; Barbieri et al., 2014; Wang and Yeung, 2017). Bone defects can be divided into small and large. Small bone defects without infection can achieve self-healing after debridement to remove nonviable tissues. At present, the main treatment methods for large bone defects are autologous, allogeneic, or bone graft substitutes (Van der Stok et al., 2011). Severe bone defects may lead to disability, affect the individual’s ability to live and work, and cause serious social and economic burdens. Bone defect repair is one of the most common regenerative surgeries, with more than 200,000 bone transplants performed worldwide every year (Beuriat et al., 2019).

The most important factor to be controlled in the process of bone substitute implantation is the risk of infection, because the body responds to the interstitial environment around the implanted biomaterial as a fibrous inflammatory area with incomplete immune function, which is vulnerable to bacterial invasion (Campoccia et al., 2013). For elective surgery, the infection rate ranges from 0.7 to 4.2%, and even as high as 30% in the case of third-degree open fractures (Clauss et al., 2010). Bone tissue infections are difficult to treat because they are located deep in the tissue and depend to some extent on the microorganisms involved. In addition, bone tissue necrosis may occur after infection or trauma, resulting in the body treating implants as foreign materials and leading to persistent, recurring infection (Calhoun et al., 2009). Patient healing time is prolonged, and repeat surgery may even be required.

In clinical settings, biomaterial-related infections are generally treated with systemic antibiotics, but high doses of antibiotics produce side effects and may lead to drug resistance in the host and bacteria. Hence, in recent years, research has focused on developing synthetic bone substitutes—biomaterials that are chemically modified in the synthesis process to meet specific biological needs. Because the skeleton is composed of a hydroxyapatite matrix and many trace elements, biomolecules, and cells, materials with complex chemical compositions are more suitable as bone substitutes than simple, pure materials (Ryan et al., 2019). Metal ions have attracted extensive attention because they do not cause drug resistance and can be delivered locally at the implantation site (Goudouri et al., 2014).

As a trace element in the human body, Cu not only has antibacterial properties and a variety of biological functions but also can promote angiogenesis (Gérard et al., 2010; Rath et al., 2016). Compared with growth factors, Cu has multiple advantages. First, it does not degrade in conventional processing and can remain stable under harsh conditions such as high temperature. In addition, copper has certain useful physical and chemical properties, including variable porosity, good mechanical strength, and crosslinking (Goh et al., 2014). It therefore could be used with other inorganic ions to generate new intelligent biomaterials that simulate the bone microenvironment. Because Cu has high antibacterial properties and low cytotoxicity, it has become a promising dopant that can confer the functional properties of bone and cartilage repair by guiding cell behavior and changing the physicochemical properties of biomaterials (Heidenau et al., 2005).

This review focuses on the application of copper combined with biomaterials in bone tissue and the associated antibacterial mechanisms. We also introduce the application of copper-based biomaterials with antibacterial and osteogenic properties in the prevention and treatment of bone infection.

## The Importance of Copper in Bone Tissue

Copper is the third most abundant essential trace element in animals and humans, surpassed only by iron and zinc. It is well known that copper is involved in the growth and maturation of many tissues and proteins, especially bone collagen (Opsahl et al., 1982; Bernabucci et al., 2013). It also can promote bone development through a catalytic metabolic process that plays an important role in human bone growth and bone mass maintenance (Rodríguez et al., 2002; Tomaszewska et al., 2017). Many studies have shown that copper is very important for bone mineralization and osteoblasts (Danks, 1981; Lowe et al., 2002). As a significant cofactor of enzymes involved in the synthesis of various bone matrix components, copper can, for example, regulate the crosslinking formation by lysyl oxidase in connective tissue, promote the synthesis of collagen and keratin, and normalize the deposition of calcium and phosphorus to form bones on fibrils. (Linder and Hazegh-Azam, 1996).

Due to the important role of Cu in bone metabolism, severe Cu deficiency will lead to bone abnormalities. In this context, the World Health Organization, the American Institute of Medicine (IOM), the European Food Safety Agency, and other national and international organizations have issued dietary standards for Cu intake to maintain good health (Tuttle et al., 2019). According to the recommendations of the IOM, the copper intake for healthy adults is 0.9 mg/day (Trumbo et al., 2001). Lack of copper in the body can lead to a variety of diseases, such as occipital angle syndrome, distal motor neuropathy, cardiovascular disease, and Menkes disease, the last of which may cause neuronal degeneration, abnormal connective tissue, abnormal lightening of hair color, and fragile bones (Wazir and Ghobrial, 2017).

In the field of tissue engineering, the use of copper can stimulate the biological characteristics required for endothelial cell proliferation during wound healing and promote the differentiation of mesenchymal stem cells into osteoblasts by upregulating the gene expression of vascular endothelial growth factor (Klatte-Schulz et al., 2012). Cu has been shown to differentiate and proliferate bone marrow mesenchymal stem cells obtained from postmenopausal women, increasing their ability to differentiate into bone and adipogenic lineages. Thus, copper has attracted attention because of its importance for bone tissue.

## Antibacterial Action of Copper-Based Biomaterials

In 2008, The United States Environmental Protection Agency recognized copper as a metallic antimicrobial effective against many disease-causing bacteria (Copper Development Associ, 2008). Studies have confirmed that copper has potential antibacterial activity against clinically relevant bacteria (Liochev and Fridovich, 2002). Infection is a matter of great concern in the medical field, so growing numbers of researchers in the field of biomaterials are focusing on the development of new copper-containing biomaterials, especially for orthopedics. Copper is added to different types of biomaterials, such as calcium phosphate bioceramics, biomedical ceramics, bioactive glass, biocomposites, and coating/alloy metals (titanium-based, stainless steel, and cobalt-based alloys) for research and antibacterial experiments.

Copper’s modes of action are highly diverse, depending on the surrounding environment, and it operates through a variety of mechanisms. Antibacterial properties of copper arise from changes in the conformational structure of nucleic acids and proteins, as well as interference with oxidative phosphorylation and osmotic balance. One of the most important modes is based on the redox properties of copper. Under aerobic conditions, copper can change its oxidation state and exchange electrons with acceptor or donor groups (such as oxygen and sulfur residues). Copper can exert its antibacterial effect directly by reacting with biomolecules or indirectly by activating oxygen species. In the latter case, copper catalyzes the production of reactive oxygen species (ROS) through a Haber–Weiss reaction similar to Fenton’s reaction (Grass et al., 2011). ROS themselves are extremely reactive when formed and quickly cause damage to surrounding biomolecules, particularly proteins, membranes, and DNA (Nandakumar et al., 2011).

Nandakumar and co-authors demonstrated the interaction of copper with proteins using a quantitative proteomics approach to identify the differential proteome profiles of *Escherichia coli* cells after exposure to metallic copper surfaces. Their results indicated that of the 509 proteins identified, 110 were differentially expressed after sub-lethal exposure, whereas 136 had significant differences in their abundance levels after lethal exposure to copper compared to unexposed cells (Ueda et al., 1980). The bactericidal potency of copper towards several bacteria with different cell envelope structures (*Streptococcus lactis*, *E. coli*, and *P. aeruginosa*) was found to be similar. The physical properties of a membrane are largely determined by its lipid composition, an important factor being the degree of fatty acid unsaturation. Cu^2+^ can interact with the SH-group, causing membrane damage and deactivation. Cu^2+^ also has a specific affinity for DNA and can bind to and disorder helical structures by crosslinking within and between strands (Wilks et al., 2005). In various studies, it was difficult to conduct antibacterial tests under the same conditions (that is, using the same medium and the same substance concentration in the medium), so it is impossible to know the specific copper release mechanism that led to the observation results. Nevertheless, it appears clear that the efficacy of contact killing was increased by higher copper content in alloys (Elguindi et al., 2009), higher temperature (Michels et al., 2009), and higher relative humidity (Wilson, 2018).

**FIGURE 1:**
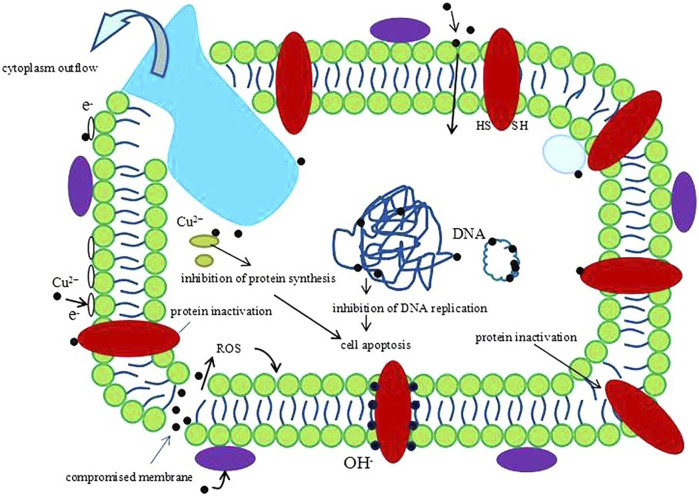
A schematic image of one hypothesis about the antibacterial mechanism of Cu^2+^. Reprinted with permission from ref. (Liu et al., 2016) (Copyright 2016; Nature Publishing Group).

Metallic biomaterials such as titanium, cobalt and tantalum are widely used in orthopedics because of their good biocompatibility, unique mechanical properties and wear resistance. However, these biomaterials are limited in their practical application in bone tissue engineering due to their poor photocatalytic antibacterial properties *in vivo*. Copper is a well-known candidate with excellent bacteria-killing ability. It is very important to introduce a proper amount of copper into titanium and other metal materials to achieve copper alloy with excellent antibacterial performance and good biocompatibility for inhibiting bacterial infection in bone implants. (Sehmi et al., 2015). In 2016, Liu et al. revealed the antimicrobial/antibiofilm activities of Ti–Cu alloy against orally specific bacterial species. From what is known so far, the bactericidal steps can be summarized as follows. First, Cu is released from a Cu-doped surface material. After bacteria are exposed to copper ions, the permeability of their cell membranes changes, leading to a loss of cytoplasmic content. Next, ROS are produced, causing further cell damage by interacting with proteins and lipids, and finally, DNA is degraded. Consequently, the bacteria cannot breathe, eat, digest, or produce energy, and cell death ensues (Figure 1) (Liu et al., 2016).

The existing form of copper in biomaterials includes two main types: 1) copper ion and 2) copper metal, both which show excellent antibacterial properties, angiogenic and osteogenic ability. To date, numerous studies have investigated their mechanisms for the antibacterial action. For the former, when bacteria are exposed to copper ions, the permeability of cell membrane could be changed, resulting in content leakage and cell lysis. Besides, copper ions inhibit protein synthesis and DNA replication. As for latter, in addition to releasing copper ions to fight bacteria, it reacts with biological molecules on bacteria, and can also produce reactive oxygen species (ROS) to damage bacteria. Both copper metal and copper ion have been widely used in bone tissue engineering due to their excellent properties, however they suffer from high toxicity. So reducing the toxicity of copper-based biomaterials remains a great challenge (Karekar et al., 2019).

## Application of Copper-Based Biomaterials

### Copper-Based Antibacterial Scaffolds

In bone tissue engineering, scaffold biomaterials are a promising method for treating bone defects and bone infections, providing a temporary framework for the integration of new bone into the defect area. The scaffold biomaterial gradually degrades, being replaced by new bone tissue until the bone repair process is completed. In treatment practice, bone-promoting and antibacterial ingredients are added to support bone remodeling. Studies have found that Cu plays an important role in bone growth, and a lack of Cu can cause bone abnormalities and decreased bone strength. Doped copper scaffold biomaterials that can promote bone healing and resist infection thus have an important potential role in bone tissue engineering.

Wu et al. prepared a Cu-containing bioactive glass (BG) scaffold with interconnected macropores and an ordered mesoporous structure. The scaffold enhanced angiogenesis and continuously release drugs, which significantly inhibited bacterial survival (Wu et al., 2013). Subha N. Rath et al. explored the biocompatibility and biological activity of Cu^2+^-doped BG scaffolds for bone marrow mesenchymal stem cells (BMSCs) and human dermal microvascular endothelial cells and found the scaffold was effective for BMSCs and not toxic. They also found that Cu^2+^ induced BMSCs to secrete vascular endothelial growth factor and thereby enhanced angiogenesis (Rath. et al., 2014). Yi-Fan Goh et al. synthesized BG doped with Cu and Ag, finding that Cu-doped BG exhibited longer ion release, making it a potential candidate for sustained long-term antibacterial performance (Goh et al., 2014). Mohamed Abudhahir et al. prepared biocomposite scaffolds of polycaprolactone, wollastonite, and Cu ions using electrospinning technology. The synthesized PCL/Cu-Ws scaffolds had good biocompatibility with mouse mesenchymal stem cells. In the presence of osteogenic mediators, the scaffold enhanced the expression of osteoblast-specific marker genes, indicating it had osteoconductivity. It also had a strong antibacterial effect on *Staphylococcus aureus* and *E. coli* (Abudhahir et al., 2021). Cu has also been doped into BG with osteoinductivity, biocompatibility, and biodegradability, then combined with a porous 3D collagen scaffold (CuBG-CS) to prepare a biological scaffold for osteomyelitis treatment. This not only promoted bone formation and angiogenesis but also inhibited *S. aureus* (Ryan et al., 2019).

**FIGURE 2:**
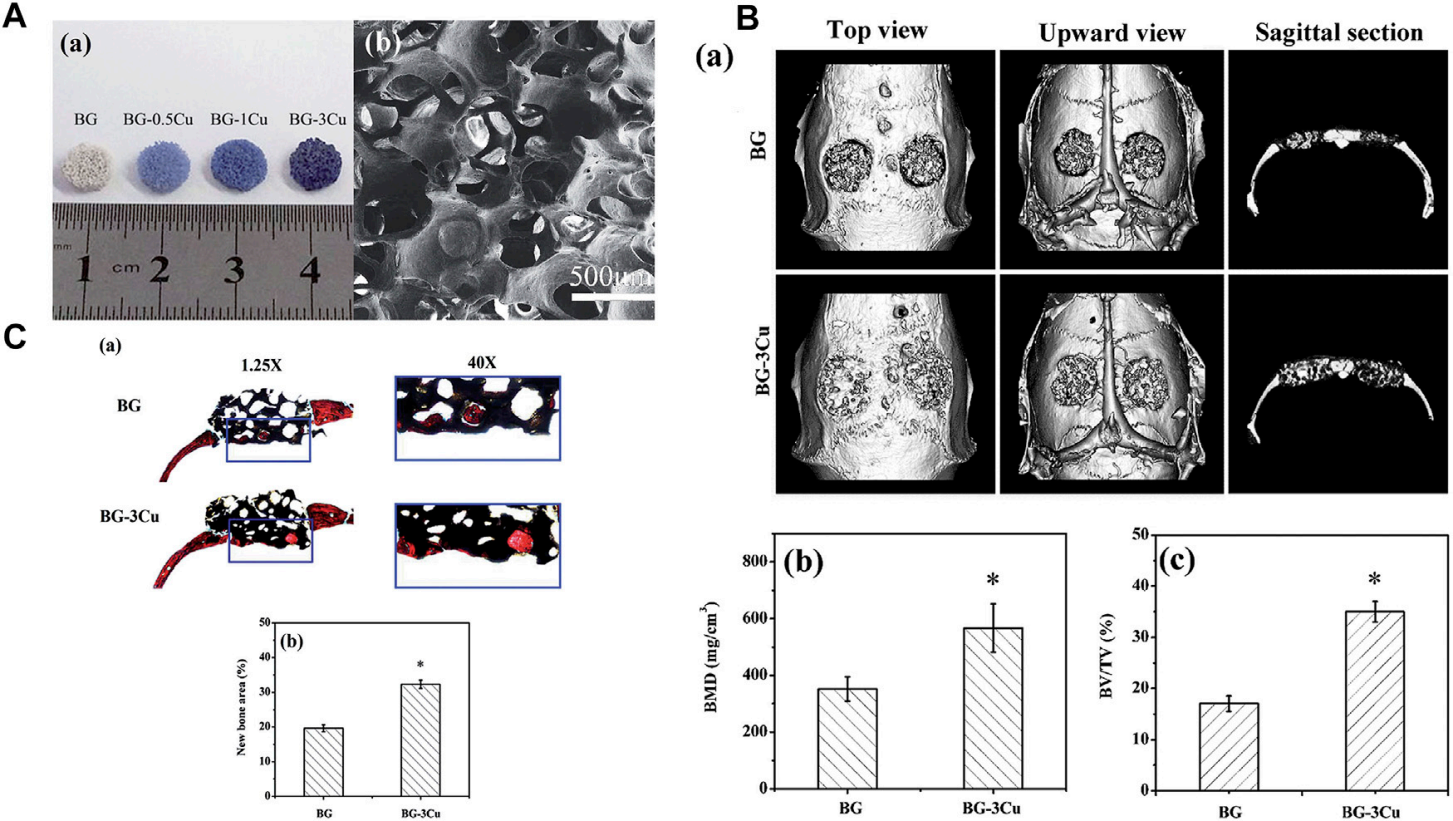
**(A)** Optical images and SEM images of the scaffolds were obtained. **(B)** Microct evaluation of skull defects and unfilled bone regeneration in rats implanted with BG-3Cu and BG stents 8 weeks after implantation. **(C)** Transmitted light images of stained sections of rat skull defects implanted with BG-3Cu and BG scaffolds and unfilled defects 8 weeks after implantation. Reprinted with permission from ref. (Wang et al., 2014). (Copyright 2014; The Royal Society of Chemistry).

Borate-based BGs are attracting attention as alternatives to silicate BGs because they more easily form porous scaffolds and degrade more quickly than silicate BGs. As shown in Figure 2, wang et al. used a polymer foam replication technique to prepare Cu-doped borate glass scaffolds and found they had better bone regeneration and angiogenesis capabilities than undoped scaffolds (Wang et al., 2014). Subsequently, the effect of Cu doping on the properties of borosilicate BG was studied, and it was found that Cu ion doping made the glass network more stable, resulting in a lower ion release rate during the degradation process. At the same time, Cu ions have the potential for angiogenesis, so this copper-doped bioactive borosilicate glass scaffold material has good prospects for use in bone tissue engineering (Wang et al., 2016).

Ethylene-vinyl acetate (EVA) copolymer has excellent mechanical properties, good biocompatibility, and high adhesion but no antibacterial ability and needs further modification. Teno et al. synthesized a CuNP-modified EVA nanocomposite material. The CuNP doping gave it antibacterial ability against DH5α *E. coli* and did not cause structural changes to or thermal degradation of the polymer (Teno et al., 2019).

As a common organic scaffold, PCL has the advantages of good biocompatibility, slow degradation rate, and low price. Mixing PCL with BG and copper metal nanoparticles can yield a nanofiber scaffold material that is nontoxic to fiber cells and has osteogenic potential (Akturk et al., 2021). Karuppannan et al. added CuO nanoparticles to PCL/gel fibers using a green synthesis method. The prepared CuONF had good mechanical properties and hydrophilicity and sustained *in vitro* drug release for 48 h. At the same time, it had an antibacterial effect on wound pathogenic bacteria and supported the growth of fiber cells, which are effective in wound care (Karuppannan et al., 2021).

### Copper-Based Antibacterial Hydrogels

**FIGURE 3:**
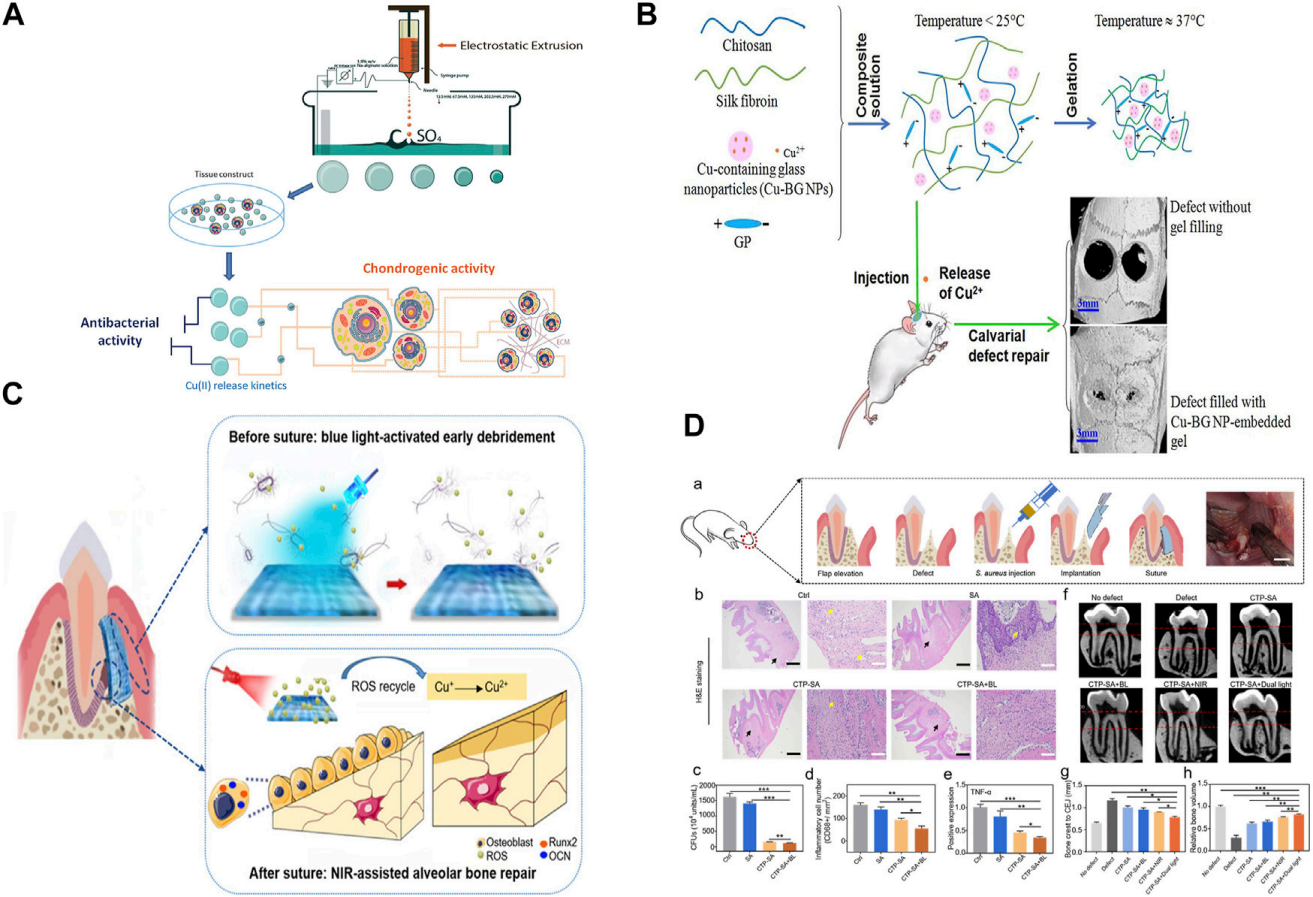
**(A)** Synthesis and potential biomedical applications of copper alginate microspheres. **(B)** Development of a novel injectable hydrogel and its osteogenic application. **(C)** Schematic diagram of surgical construction of CTP-SA and GTR. **(D)** *In vivo* function of CTP-SA in GTR surgery. Panel **(A)** reprinted with permission from ref. (Madzovska-Malagurski et al., 2016) (Copyright 2016; IOP Publishing Ltd.). Panel **(B)** reprinted with permission from ref. (Bassett et al., 2014) (Copyright 2019; Elsevier). Panel **(C)** and panel **(D)** reprinted with permission from ref. (Xu et al., 2020) (Copyright 2020; American Chemical Society).

Hydrogels with high oxygen permeability, water absorption and expansion, good biocompatibility, and other distinctive properties have been widely used in the biomedical field (Kopeček and Yang, 2007; Wang et al., 2019; Gao et al., 2020). Alginate saline gel produced by ion crosslinking of alginate with other ions has been applied to soft tissue implants and wound dressings because it requires only mild preparation conditions and has other attractive properties (Queen et al., 2004; Lee and Mooney, 2012; Huang et al., 2021). However, alginate saline gel has some limitations in practical applications because it has no biological activity or antibacterial properties. Levic et al. prepared Cu-alginate hydrogels in the form of microbeads by adding copper to alginate by electrostatic extrusion. The results showed that microspheres loaded with ∼100 μmol g^−1^ Cu (II) had high antibacterial activity against *E. coli* and *S. aureus*. Microspheres with low Cu(II) (∼60 μmol g^−1^) content released Cu(II) slowly enough to induce the proliferation of calf chondrocytes and promote chondrogenesis (Figure 3A) (Madzovska-Malagurski et al., 2016).

The use of metal ions, especially copper ions, in medical applications may present serious problems of acute local and systemic toxicity and lead to narrow therapeutic wounds. Hence, it is critical to achieve precise control and sustained release of copper ions in bone tissue applications. David et al. used alginate as a carrier for copper storage and as a copper ion diffusion barrier, doping copper materials into alginate brine gel microspheres through *in situ* mineralization and subsequent crosslinking with calcium. This method not only increased the amount of copper supported by alginate saline gel but also controlled the rate and duration of ion release. By altering the formulation of alginate saline gels containing both ionic and mineral copper, the total copper content was changed and the release time of Cu^2+^ could be adjusted from 5 to 32 days (Figure 3B) (Bassett et al., 2014).

Injectable hydrogels are also being developed to automatically match bone defects better (Wu et al., 2019). Wang et al. prepared sodium alginate hydrogel doped with cuprous oxide (Cu_2_O) nanoparticles and polydopamine (PDA)-coated titanium dioxide (TiO_2_@PDA) nanoparticles to form a composite (CTP-SA) for guided tissue regeneration to treat periodontitis. The increased release of Cu^2+^ and the photothermal effect of TiO_2_@PDA when combined with NIR irradiation endowed the composite with osteogenic properties, which were beneficial for alveolar bone repair. This kind of bone implant material, which simultaneously enables dual customization of shape and function, has the potential for multiple applications in biomedicine (Figures 3C,D) (Xu et al., 2020).

### Copper-Based Antibacterial Bone Cements

Bone cement based on polymethyl methacrylate (PMMA) is a unique biological material with the advantages of easy processing and good biological stability (Vaishya et al., 2013; Wekwejt et al., 2019). Antibiotic-loaded bone cement can greatly reduce the adhesion and proliferation of bacteria, but most antibiotics are not effective for continuous treatment after surgery, and long-term use can lead to the production of drug-resistant bacteria. It is therefore very important to develop new bone cements with excellent antibacterial properties. Metal nanoparticles such as Cu and Ag have good bactericidal properties and can be combined with PMMA to improve the bactericidal activity of bone cement. This is mainly due to the release of free metal ions, which cause direct damage to cell membranes and are absorbed by the cells, then produce reactive oxygen species. These processes can lead to bacterial death (Tamayo et al., 2016; Das et al., 2017; Burdușel et al., 2018; Darder et al., 2020).

Wekwejt et al. studied the bactericidal performance of PMMA modified with gentamicin, nanosilver, and nanocopper and found that compared with PMMA modified with gentamicin, PMMA modified with nanocopper had a higher bactericidal effect and better anti-biofilm performance but at the same time reduced the vitality of pulp stem cells (Miola et al., 2018). Miola et al. designed a new type of Cu-doped bioactive glass powder composite cement, added it to PMMA-based cement with different viscosities, and characterized the prepared composite materials in terms of their biological activity, mechanical properties, and antibacterial properties. The results showed that the glass powder was evenly distributed on the surface of PMMA, and the composite cement had good biological activity and obvious antibacterial effect on *Staphylococcus epidermidis* strains (Uriu-Adams and Keen, 2005). PMMA itself is not degradable and has low adhesion ability, so modifications are very important for improving its biocompatibility and antibacterial properties. We summarized the application of copper-based biomaterials in bone tissue in Table 1, including antibacterial scaffolds, antibacterial hydrogels and antibacterial bone cement.

**TABLE 1:** Representative examples of copper-based antibacterial biomaterials.

Copper-based biomaterials	Component	Type of Cu	Result	Application	References
Antibacterial scaffolds	Bioactive glass (BG)	Cu^2+^	Release of ibuprofen and antibacterial	Infection of regenerated bone	Wu et al. (2013)
		Cu^2+^	Good bioactivity in bone marrow tissue	Bone scaffold	Rath et al. (2014)
			Cu	*In vitro* antibacterial	Bone regeneration	Goh et al. (2014)
	Polycaprolactone (PCL)	Cu^2+^	*In vitro* antibacterial	Bone tissue engineering	Abudhahir et al. (2021)
		Cu	Have a positive effect on fibroblast cell	Bone tissue engineering	Akturk et al. (2021)
				CuO	*In vitro* antibacterial	Antibacterial wound dressing	Karuppannan et al. (2021)
		Collagen scaffold (CS)	Cu^2+^	Antibacterial and enhancing bone healing	Infection osteomyelitis	Ryan et al. (2019)
		Borate	CuO	Stimulate angiogenesis and regenerate bone	Healing bone defects	Wang et al. (2014), Wang et al. (2016)
		Ethylene-vinyl acetate (EVA)	CuNPs	*In vitro* antibacterial	Antibacterial	Teno et al. (2019)
Antibacterial hydrogels	Alginate	Cu^2+^	*In vitro* antibacterial	Antimicrobial wound dressings and tissue engineering scaffolds	Queen et al. (2004), Lee and Mooney (2012), Madzovska-Malagurski et al. (2016), Huang et al. (2021)
			Cu^2+^	Good bioactivity	Bone tissue engineering alternatives	Bassett et al. (2014)
		TiO_2_@PDA	Cu_2_O	Antibacterial and osteogenic	Periodontitis	Xu et al. (2020)
Antibacterial bone cements	Polymethyl methacrylate (PMMA)	Cu	*In vitro* antibacterial	Antibiofilm and bone tissue engineering	Uriu-Adams and Keen (2005), Tamayo et al. (2016), Das et al. (2017), Burdușel et al. (2018), Miola et al. (2018), Darder et al. (2020)
		Cu^2+^			

## Conclusion and Outlook

This paper reviews the antibacterial properties and mechanism of copper-based biomaterials, as well as their application in bone tissue engineering, including antibacterial scaffolds, antibacterial hydrogels and antibacterial bone cement. Copper is an essential trace element involved in various physiological mechanisms of human beings. Many studies have shown that copper-based biomaterials have excellent antibacterial properties, angiogenesis and osteogenesis. So copper can be used as a promising dopant in combination with other biomaterials to effectively treat pathogenic infections in bone tissue, providing an interesting alternative to the extensive use of antibiotics and avoiding the development of antibiotic resistance. With regard to the role of copper in osteogenesis, it seems difficult to draw conclusions about effects on specific targets, since osteogenesis involves a very complex mechanism associated to multiple factors. Although beneficial therapeutic effects have been proved, the exact mechanism of action is still unclear, so it is difficult to accurately study and describe the effect of copper-based biomaterials on this physiological mechanism. The fact that more research is needed to clearly understand how copper interacts with the physiological processes of osteogenesis has important implications for orthopaedic applications.

The copper-based biomaterials show good antibacterial properties and low infection rates after bone implantation, copper deficiency and excessive accumulation however can lead to serious diseases and certain toxicity (Shim and Harris, 2003). For adults, the tolerable upper limit is 10 mg/day and impairments occur above this level (Shenkin, 2010). Therefore, the biotoxicity of copper-based biomaterials should be evaluated before it is used in bone tissue engineering. It has been demonstrated that the toxicity of copper-based biomaterials is reduced by optimizing copper doping concentration, releasing curve or reducing metal particle size (Chen et al., 2021). At the same time, biocompatibility and antibacterial ability must also be achieved. At present, most experiments evaluate the toxicity of copper-based biomaterials by testing them with bone marrow stem cells, osteoblasts, and other cells, and using *in vivo* experiments in small animals (Prajapati et al., 2020). However, these do not simulate the human body’s true response to toxicity, so more clinical trial results are needed.
